# Factors associated with recruitment to randomised controlled trials in general practice: protocol for a systematic review

**DOI:** 10.1186/s13063-019-3354-z

**Published:** 2019-05-10

**Authors:** Keith R. Moffat, Paul Cannon, Wen Shi, Frank Sullivan

**Affiliations:** 1 Population and Behavioural Science Division, School of Medicine, Medical & Biological Sciences, North Haugh, St Andrews, UK; 2 Information Services, University of Glasgow, Hillhead Street, Glasgow, G12 8QE UK

**Keywords:** recruitment, general practice, randomised controlled trial

## Abstract

**Background:**

Randomised controlled trials (RCTs) are frequently unable to recruit sufficient numbers of participants. This affects the trial’s ability to answer the proposed research question, wastes resources and can be unethical. RCTs within a general practice setting are increasingly common and similarly face recruitment challenges. The aim of the proposed review is to identify factors that are associated with recruitment rates to RCTs in a general practice setting. These results will be used in further research to predict recruitment to RCTs.

**Methods/design:**

The electronic databases Medline, EMBASE, Cochrane Database of Systematic Reviews, NTIS and OpenGrey will be searched for relevant articles with no limit on the date of publication. *BMC Trials* will be manually searched for the past 5 years. Both quantitative and qualitative studies will be included if they have studied recruitment within a general practice RCT. Only English language publications will be included. Screening, quality assessment and data extraction will be conducted by two review authors not blinded to study characteristics. Disagreement will be resolved by discussion and the involvement of a third review author if required. A narrative synthesis of the studies included will be performed.

**Discussion:**

The review will, for the first time, systematically synthesise existing research on factors associated with recruitment rates to RCTs in general practice. By identifying research gaps to be prioritised in further research, it will be of interest to academics. It will also be of value to clinical trialists who are involved in the complex task of improving trial recruitment. Our team will use the findings to inform a prediction model of trial recruitment using machine learning.

**Systematic review registration:**

PROSPERO, CRD42018100695. Registered on 03 July 2018.

**Electronic supplementary material:**

The online version of this article (10.1186/s13063-019-3354-z) contains supplementary material, which is available to authorized users.

## Background

Randomised controlled trials (RCTs) require a sufficient number of participants to be adequately powered. This is necessary for the trial to answer the particular research question. The significant difficulties in recruiting sufficient numbers of participants have been well established [1–3]. A review of all 151 RCTs funded by the Health Technology Assessment Programme of the UK National Institute for Health Research between 2004 and 2016 showed that only 40% of RCTs were able to recruit 100% of their original target, 63% were able to recruit 80% of their original target and around one third of RCTs extended their recruitment period to increase recruitment [1]. Whilst this review does not report actual recruitment as a percentage of the target sample for those studies in general practice, other studies have similarly described difficulties in recruiting individuals to general practice studies [4–6]. As a result of the challenges in recruiting participants, RCT recruitment has been set as a priority research area [7].

This systematic review will focus on recruitment rates to RCTs in general practice. The reason for this is that for many nations, care delivered in the community is the intended paradigm of health care [8, 9]. Given that health care will increasingly be delivered in the community, we are interested in specifically researching RCTs in a general practice setting. Research findings from RCTs that have studied only a hospital setting cannot be assumed to translate to a community setting where most care is delivered. Therefore, it is important that we have a robust evidence base of treatment effectiveness for people in the community. This requires research into interventions that are specifically delivered in the community and, as such, a research priority is recruitment in a general practice setting. This has been recognised by the development of practice-based research networks internationally [10–12].

A relevant area for our own research is that of predictive modelling of recruitment to trials and the call for better models in predicting recruitment [13]. This approach is supported by a recent priority setting exercise by the James Lind Alliance (PRioRiTy) for research into RCT recruitment. A major priority identified for research into RCT recruitment included: “What are the best ways to predict recruitment rates to a randomized trial and what impact do such predictions have on recruitment?” [14].

We aim to develop a predictive model of RCT recruitment rates using machine-learning methods. So that the appropriate data are used for this model, it is necessary to identify the factors that have hitherto been identified as being associated with recruitment to RCTs. Although this will be of broader interest, we will be using the outcomes from this systematic review to select appropriate datasets for our model. It is important to state that the factors identified in the systematic review need not have been shown to *predict* or *affect* recruitment, since machine-learning algorithms will determine predictability during development.

Other systematic reviews in this research area include the Cochrane reviews by Treweek et al*.*, which have focused upon interventions to improve recruitment to RCTs [15–17]. Whilst this is clearly important, for our purposes this would not be sufficient because they identify factors associated with recruitment in the context of an intervention. For example, there are studies that retrospectively investigate reasons for poor recruitment, which is of value for our purpose but is not interventional by type [18]. Additionally, we expect there will be interventional studies aiming to improve recruitment rates but that are not randomised or quasi-randomised, which would have been omitted by Treweek et al.’s selection criteria. An ongoing Cochrane qualitative evidence synthesis of factors that impact recruitment to randomised trials [19] is similarly not broad enough for our purposes, since it will be including only studies investigating the participant perspective in recruitment. As such, it will not investigate the perspective of the recruiter (personal correspondence, 12 January 2018), e.g. general practices as a unit or general practitioners. This study will include both recruiters and participants. Furthermore, none of these reviews is focused on the area of general practice, as they look at recruitment more widely.

## Methods/design

The protocol has been developed according to the PRISMA guidelines for systematic review protocols (PRISMA-P) [20]. The completed PRISMA-P checklist for this protocol is given as Additional file 1. The protocol has been registered with PROSPERO (registration number CRD42018100695), which can be accessed via the following webpage: https://www.crd.york.ac.uk/PROSPERO/display_record.php?RecordID=100695

### Aim

The aim of this systematic review is to identify factors associated with recruitment of individuals and practices to RCTs in general practice.

### Eligibility criteria

The following criteria will be used in selecting studies for the systematic review:

1. Study design
  Any primary study design that investigates recruitment to RCTs in general practice will be included. RCTs based in general practice will not themselves be included unless they have also investigated recruitment. Qualitative and quantitative studies will be included.
2. Participants
  Studies of any types of participant will be included. Studies will be included both where the focus is on recruitment of practices to RCTs as well as individual participants.
3. Interventions
  Studies of any types of intervention that have targeted recruitment will be included.
4. Comparators
  Studies of any types of comparator will be included. Interventional study designs are likely to compare to the usual recruitment method. Observational studies may report variations based on sociodemographic variables or system differences, e.g. research networks and payments.
5. Outcomes
  Only studies where at least one outcome has focused on recruitment will be included. Examples of such outcomes include the number of participants recruited, percentage of recruitment target achieved and time to first participant recruited. The different recruitment outcomes will be collated and described.
6. Setting
  Only RCTs investigating recruitment within a general practice setting will be included. General practices can be involved in recruitment in two main ways. The first is as the locus of the intervention such that recruited participants are randomised to an intervention based in the practice. The second is where the practice is used to recruit participants to a trial that is not based within a general practice setting. Only studies in the former setting will be included.
7. Language
  Only studies in English will be included.

### Information sources

The following databases were searched for relevant studies:

- Ovid MEDLINE(R) In-Process & Other Non-Indexed Citations and Ovid MEDLINE, 1946 to present via OvidSP
- Embase 1947-Present, updated daily via OvidSP
- Cochrane Central Register of Controlled Trials (CENTRAL): Issue 4 of 12, April 2018 & Cochrane Database of Systematic Reviews (CDSR): Issue 5 of 12, May 2018 via the Cochrane Library

All databases were searched on 23 May 2018. In addition, the websites OpenGrey (http://www.opengrey.eu/) and the National Technical Reports Library (https://ntrl.ntis.gov/NTRL/) were searched on 1 June 2018. The journal *BMC Trials* will be manually searched for the preceding 5 years (from the 1st of June 2013 to the 1st of June 2018). Experts in the field of trial recruitment were asked to suggest further important articles for inclusion.

### Search strategy

The search strategy was supported by a health information specialist with systematic review experience (PC). The search strategy used both text words and relevant indexing for controlled or multicentre trials, selection and recruitment, and general practices and practitioners. Citation and bibliographic searches will be conducted on all included studies to identify additional relevant studies. The search will be updated towards the end of the review. The full search strategy is given in Additional file 2.

### Study records

Literature search results will be exported to the systematic review software DistillerSR. Based on the inclusion and exclusion criteria, the review team will develop screening questions and forms, which will be tested by a calibration exercise prior to implementation. These will be uploaded to DistillerSR along with citation abstracts and full articles. All members of the review team not familiar with DistillerSR or the subject area will receive training. A PRISMA diagram [21] will be completed to show the numbers of studies selected at different levels of assessment.

### Selection process

Titles and abstracts of studies found using the search strategy and from other sources will be independently screened by two reviewers (KM and WS). The full text of those studies that potentially meet the inclusion criteria will be retrieved and independently assessed for eligibility by two reviewers. Any disagreements over the eligibility of studies will be resolved through discussion with a third reviewer (FS). Reasons for excluding studies will be recorded. The reviewers will not be blinded to the journal titles, study authors or institutions.

### Data collection process

Standardised forms will be used to extract data into DistillerSR. Two review authors (KM and WS) will extract data independently from the studies included, and conflicts will be resolved through discussion. Any disagreements will be resolved with a third reviewer (FS). Extracted data will include demographic information, methodology and outcomes as well as measured recruitment metrics. Study authors will be contacted if any relevant data are incomplete.

### Outcomes

The outcome will be study recruitment. There are many ways of measuring this, including numbers, proportions and rates. These will be captured during data extraction. Additionally, recruitment outcomes of both practices and individuals will be extracted.

### Risk of bias

Two reviewers will independently assess the risk of bias. This will be assessed at the study level. The Cochrane collaboration tool for assessing the risk of bias [22] will be used for RCTs and the relevant CASP checklist will be used for non-RCT studies [23]. This information will be tabulated along with the corresponding studies and will inform our discussion of the quality of the evidence base on this topic.

### Data synthesis

A narrative synthesis of the included studies will be performed. The information will be presented in text and tabular format to summarise the characteristics and results of the included studies as well as to explain the outcomes within and between the studies. Guidance from the Centre for Reviews and Dissemination will inform the synthesis [24]. It is anticipated that included studies will have a high risk of bias due to their design. For this reason, all studies will be included in the synthesis with an appropriate discussion of their limitations.

## Discussion

This will be the first time that a systematic review has synthesised factors associated with recruitment to RCTs in general practice. In doing so, it will comprehensively identify recruitment factors that should be considered by both those conducting trials and also trial researchers. The quality of the included studies will inform further research in trial recruitment by identifying research gaps reflected by studies of poorer quality. Finally, the findings will directly inform a model that our team is developing to predict recruitment to RCTs in general practice using machine learning methods.

## Additional files

Additional file 1: PRISMA-P 2015 checklist. (DOCX 32 kb)

Additional file 2: Search strategy (PDF 212 kb)
