# Supplementary material for: Factors associated with recruitment to randomised controlled trials in general practice: protocol for a systematic review
Source: Trials. 2019 May 10;20:266. doi: 10.1186/s13063-019-3354-z (PMC6511135; doi:10.1186/s13063-019-3354-z)
Supplement: Supplementary file 2 — Search strategy (PDF 212 kb) [file 13063_2019_3354_MOESM2_ESM.pdf]

Source: Ovid MEDLINE(R) In-Process & Other Non-Indexed Citations and Ovid MEDLINE
Interface: OvidSP
Database coverage dates: 1946 to Present
Search date: 23 May 2018
Retrieved records: 2565
Search strategy:

1 exp Clinical Trials as Topic/ (314264)
2 Multicenter Studies as Topic/ (16891)
3 ((clinical or controlled or multicenter or multicentre or multi-center or multi-centre) adj2 (trial? or study or studies or research)).tw. (767858)
4 or/1-3 (955516)
5 Patient Selection/ (59037)
6 Patient Participation/ (22511)
7 Personnel Selection/ (12194)
8 (participat* or recruit* or enrol*).tw. (968575)
9 or/5-8 (1043466)
10 exp Informed Consent/ (38811)
11 (informed adj (consent or decision* or choice*)).tw. (38569)
12 or/10-11 (67122)
13 9 or 12 (1096693)
14 exp General Practice/ (71870)
15 General Practitioners/ (6148)
16 Physicians, Family/ (15790)
17 (family adj (practitioner? or practice? or doctor? or physician?)).tw. (24864)
18 (general adj (practitioner? or practice?)).tw. (72546)
19 or/14-18 (136820)
20 4 and 13 and 19 (2565)

Source: Embase 1947-Present, updated daily
Interface: OvidSP
Database coverage dates: 1947 to present
Search date: 23 May 2018
Retrieved records: 3545
Search strategy:

1 exp "clinical trial (topic)"/ (268156)
2 ((clinical or controlled or multicenter or multicentre or multi-center or multi-centre) adj2 (trial? or study or studies or research)).tw. (1110689)
3 or/1-2 (1236479)
4 patient selection/ (84060)
5 patient participation/ (23530)
6 personnel management/ (55756)
7 (participat* or recruit* or enrol*).tw. (1461388)
8 or/4-7 (1601441)
9 informed consent/ (93436)
10 (informed adj (consent or decision* or choice*)).tw. (74374)
11 or/9-10 (120989)
12 8 or 11 (1692683)

13 general practice/ (80065)
14 general practitioner/ (89915)
15 (family adj (practitioner? or practice? or doctor? or physician?)).tw. (31843)
16 (general adj (practitioner? or practice?)).tw. (96029)
17 or/13-16 (199931)
18 3 and 12 and 17 (3545)

Source: Cochrane Central Register of Controlled Trials (CENTRAL): Issue 4 of 12, April 2018 & Cochrane Database of Systematic Reviews (CDSR): Issue 5 of 12, May 2018
Interface: Cochrane Library/Wiley Interscience
Database coverage dates: Not available
Search date: 23 May 2018
Retrieved records: CENTRAL subset = 553 & CDSR subset = 15
Search strategy:

#1 MeSH descriptor: [Clinical Trials as Topic] explode all trees 57992
#2 MeSH descriptor: [Multicenter Studies as Topic] this term only 2039
#3 ((clinical or controlled or multicenter or multicentre or multi-center or multi-centre) near/2 (trial? or study or studies or research)):ti,ab 164485
#4 #1 or #2 or #3 209112
#5 MeSH descriptor: [Patient Selection] this term only 3646
#6 MeSH descriptor: [Patient Participation] this term only 1228
#7 MeSH descriptor: [Personnel Selection] this term only 54
#8 (participat* or recruit* or enrol*):ti,ab 175407
#9 #5 or #6 or #7 or #8 178120
#10 MeSH descriptor: [Informed Consent] explode all trees 672
#11 (informed next (consent or decision* or choice*)):ti,ab 8638
#12 #10 or #11 8965
#13 #9 or #12 182907
#14 MeSH descriptor: [General Practice] explode all trees 2636
#15 MeSH descriptor: [General Practitioners] this term only 220
#16 MeSH descriptor: [Physicians, Family] this term only 488
#17 (family next (practitioner? or practice? or doctor? or physician?)):ti,ab 839
#18 (general next (practitioner? or practice?)):ti,ab 4072
#19 #14 or #15 or #16 or #17 or #18 6628
#20 #4 and #13 and #19 595

Source: OpenGrey
Interface: http://www.opengrey.eu/
Database coverage dates: Not available
Search date: 1 June 2018
Retrieved records: 230 (filtered to English language)
Search strategy:

((family NEAR/1 (practitioner* or practice* or doctor* or physician*) OR (general NEAR/1 (practitioner* or practice*)))

Source: National Technical Reports Library (NTIS)

Interface: https://ntrl.ntis.gov/NTRL/
Database coverage dates: Not available
Search date: 1 June 2018
Retrieved records: 65
Search strategy:

(("family practitioner" or "family doctor" or "family physician" or "family practice" or "general practitioner" or "general practice") and (rct or "clinical trial" or "controlled trial" or "multicenter study" or "multi-center study") and (participat* or recruit* or enrol* or "informed consent" or "informed decision" or "informed choice"))
